# Levels of Intra-specific AFLP Diversity in Tuber-Bearing Potato Species with Different Breeding Systems and Ploidy Levels

**DOI:** 10.3389/fgene.2017.00119

**Published:** 2017-09-21

**Authors:** Glenn J. Bryan, Karen McLean, Robbie Waugh, David M. Spooner

**Affiliations:** ^1^Cell and Molecular Sciences, The James Hutton Institute Invergowrie Dundee, United Kingdom; ^2^Vegetable Crops Research Unit, Department of Horticulture, United States Department of Agriculture-Agricultural Research Service (USDA-ARS), University of Wisconsin-Madison, Madison WI, United States

**Keywords:** AFLP, diversity, genebank, potato, *Solanum*

## Abstract

DNA-based marker analysis of plant genebank material has become a useful tool in the evaluation of levels of genetic diversity and for the informed use and maintenance of germplasm. In this study, we quantify levels of amplified fragment length polymorphism (AFLP) in representative accessions of wild and cultivated potato species of differing geographic origin, ploidy, and breeding system. We generated 449 polymorphic AFLP fragments in 619 plants, representing multiple plants (16–23) from 17 accessions of 14 potato taxa as well as single plants sampled from available accessions (from 3 to 56) of the same 14 taxa. Intra-accession diversities were compared to those of a synthetic ‘taxon-wide’ population comprising a single individual from a variable number of available accessions of each sampled taxon. Results confirm the expected considerably lower levels of polymorphism within accessions of self-compatible as compared to self-incompatible taxa. We observed broadly similar levels of ‘taxon-wide’ polymorphism among self-compatible and self-incompatible species, with self-compatible taxa showing only slightly lower rates of polymorphism. The most diverse accessions were the two cultivated potato accessions examined, the least diverse being the Mexican allohexaploids *Solanum demissum* and *S. iopetalum*. Generally allopolyploid self-compatible accessions exhibited lower levels of diversity. Some purported self-incompatible accessions showed relatively low levels of marker diversity, similar to the more diverse self-compatible material surveyed. Our data indicate that for self-compatible species a single plant is highly representative of a genebank accession. The situation for self-incompatible taxa is less clear, and sampling strategies used will depend on the type of investigation. These results have important implications for those seeking novel trait variation (e.g., disease resistance) in gene banks as well as for the selection of individuals for genomics studies. We also show that AFLPs, despite having been largely replaced by other marker types, is highly suitable for the evaluation of within and between accession diversity in genebanks.

## Introduction

Collections of *ex situ* crop plant germplasm have the primary function of preserving plant genetic diversity for future use through plant breeding and biological research. The measurement of genetic diversity in plant germplasm has been revolutionized by the use of molecular markers. Such molecular tools allow a quantitative, objective assessment of the amount and distribution of genetic diversity, and permit a more rational use of plant material in breeding, research and germplasm maintenance as well as informing the process by which genebank material is maintained. The molecular tools available to the genebank curator are becoming ever more sophisticated, inexpensive, and easy to use for large collections (Spooner et al., 2005).

The potato and its wild relatives (*Solanum* section *Petota*) contain 107 wild (Spooner et al., 2014) and four cultivated (Ovchinnikova et al., 2011) tuber-bearing taxa, which grow from the southern United States through Central and South America, and occupy a wide range of ecological niches (Hijmans and Spooner, 2001). They differ in many fundamental ways that could significantly affect their genetic diversity: selfing or outcrossing; diploidy, disomic polyploidy or polysomic polyploidy; and endosperm balance numbers (EBN). The EBN relates to a strong interspecific biological isolating mechanism in sect. *Petota*, due to the functioning or breakdown of the endosperm after fertilization (Hanneman, 1994), and is of great predictive value in practical breeding programs in potato. The EBN hypothesis proposes that a 2 maternal:1 paternal ratio of genes, rather than genomes, is necessary for normal endosperm development in potatoes (Johnston et al., 1980). EBNs are determined empirically relative to other EBNs, and can be modulated within a species by various methods including ploidy manipulations and bridge crosses, embryo rescue, hormone treatments, reciprocal crosses and protoplast fusion (Jansky, 2006).

Self-incompatibility in the Solanaceae arises from an RNA-based gametophytic system. A broad-scale phylogenetic study suggests that in the Solanaceae its evolutionary loss appears to be irreversible, so reducing the prospects for the persistence or speciation of taxa (Igic et al., 2003). Most diploid *Solanum* species from section *Petota* are self-incompatible (SI); only four diploid taxa reported to be self-compatible (SC): *S. chacoense*, *S. morelliforme, S. polyadenium* and *S. verrucosum*; and two additional taxa from the close outgroup section *Etuberosum* to also be SC: *S. etuberosum, S. palustre* (Hanneman, 1985; Hawkes, 1990). Polyploid *Solanum* taxa are all considered to be SC, with the exception of *S. tuquerrense* (Hawkes, 1990). Cultivated tetraploid potato *S. tuberosum* Andigenum group (from the uplands in the Andes) and Chilotanum group (lowland Chile) is strictly SC but suffers acutely from inbreeding depression, and so is considered as SI in this study.

Several large potato germplasm collections contain many of the wild and cultivated species of potato, but to date there has been only limited study of the molecular diversity among the different ploidy and breeding systems. RAPDs have been used within sect. *Petota* to measure correlations between molecular diversity and ecogeographic factors. For example, del Rio et al. (2001) and del Rio and Bamberg (2002) associated DNA marker variation with ecogeographical differences, but found no link between genetic and ecogeographic factors. del Rio and Bamberg (2004) showed significant associations between genetic parameters and ecogeographic factors, such as physical separation, latitude, longitude, and proximity to other *Solanum* species for the diploid inbreeding species *S. verrucosum* Schltdl. Bamberg and del Rio (2004) compared genetic (as measured using RAPD) variability among populations of four tuber-bearing wild species differing in breeding system, and showed marked differences in intrapopulational genetic heterogeneity (GH), as defined by a measure of average estimated heterozygote frequency. These authors report that GH values appear to be much lower in the two SC taxa surveyed (*S. stoloniferum* and *S. verrucosum*) than in two SI species (*S. jamesii* and *S. sucrense*, the latter now placed by Spooner et al. (2014) into *S. brevicaule*).

This study is aimed at the quantification of levels of marker polymorphism among individuals from 17 *ex situ* accessions, comprising 14 taxa representing all of the commonly observed ploidy levels and breeding systems in potato. Our strategy is based on a comparison of intra-accession variability with a ‘taxon wide’ estimate obtained using a synthetic population comprising a single randomly chosen individual from a representative sample of accessions of each taxon. We have used amplified fragment length polymorphisms (AFLPs) in this study due to their highly multiplex and repeatable nature and to their demonstrated homology across fairly wide taxonomic distances (Kardolus et al., 1998). In earlier studies, RAPDs were adopted but we chose not to use this marker type due to its problems with low reproducibility (Schierwater and Ender, 1993) and non-homology across unrelated germplasm. For example, a study of genetic diversity in two phylogenetically diverse species of potato (*Solanum fendleri* A. Gray = *S. stoloniferum* Schltdl. and *S. jamesii* Torr.) by del Rio et al. (1997) required different sets of RAPD primers for each species. Our choice was also influenced by the superior ‘marker index’ (the product of heterozygosity and number of individual markers produced per marker assay), robustness, and ‘transferability’ across closely related germplasm and its proven suitability for use in diverse *Solanum* germplasm (Milbourne et al., 1997; McGregor et al., 2000). Our intention is to assess whether the levels of marker diversity preserved in *ex situ* populations are roughly in accordance with what would be expected from knowledge of the respective breeding systems used. Where possible, we hope to relate these findings to previous studies of plant diversity, which were largely based on use of isozyme or RAPD markers.

## Materials and Methods

### Plant Material

Seventeen accessions of 13 species held in the Commonwealth Potato Collection (CPC) or the U.S. Potato Genebank (NRSP-6) were selected to represent the range of different ploidy levels, breeding systems, and EBNs found in section *Petota* and close outgroup section *Etuberosum* (*S. palustre*) (**Table 1**). Identifications of the accessions examined here (**Table 1**) follow Spooner et al. (2014) where *S*. ×*aemulans* was formerly identified as *S. acaule* subsp. *aemulans* (Bitter and Wittm.) Hawkes and Hjert., *S. boliviense* as *S. megistacrolobum* Bitter, *S. brevicaule* 2x as *S. sparsipilum* (Bitter) Juz. and Buk., *S. brevicaule* 4x and 6x as *S. oplocense* Hawkes 4x and 2x, and *S. candolleanum* as *S. bukasovii* Juz. Twenty-four plants per accession were grown in the glasshouse from true seed. Additionally, single plants from other available accessions of the selected species were raised to allow comparisons of levels of polymorphism within and between accessions.

**Table 1:** Potato species and accessions studied^a^, ploidy level, breeding system, endosperm balance numbers (EBN), and numbers of plants included in the study.

Taxon (code)	Acc #^b^	Ploidy	EBN	N1^c^	N2^c^
**Self-incompatible species^d^**					
*S. boliviense* Dunal (blv)	7020	2x	2	20	11
*S. brevicaule* Bitter (brc2x)	3564	2x	2	19	24
*S. brevicaule* (possibly SI see text) (brc4x)	442693	4x	4	23	7
*S. brevicaule* (possibly SI see text) (brc6x)	545908	6x	4	18	10
*S. candolleanum* Berthault (cnd)	2723	2x	2	19	14
*S. ehrenbergii* (Bitter) Rydb. (ehr)	7507	2x	1	18	3
*S. pinnatisectum* Dunal (pnt)	347766	2x	1	20	5
*S. tuberosum* L. Andigenum group 2x (tbr2x)	234012	2x	2	23	56
*S. tuberosum* Chilotanum group 4x (tbr4x)	245929	4x	4	23	11
**Self-compatible species**					
*S. acaule* Bitter (acl)	82	4x	2	18	17
*S.* ×*aemulans* Bitter and Wittm. (aem)	7004	4x	2	17	11
*S. demissum* Lindl. (dms1)	27	6x	4	18	50
*S. demissum* Lindl. (dms2)	3850	6x	4	18	50
*S. iopetalum* (Bitter) J. G. Hawkes (iop)	7028	6x	4	17	14
*S. palustre* Poepp. (pal)	7135	2x	1	16	6
*S. stoloniferum* Schltdl. and Bouchet (sto)	595472	4x	2	21	44
*S. verrucosum* Schltdl. (ver)	558488	2x	2	23	23


**FIGURE 1:**
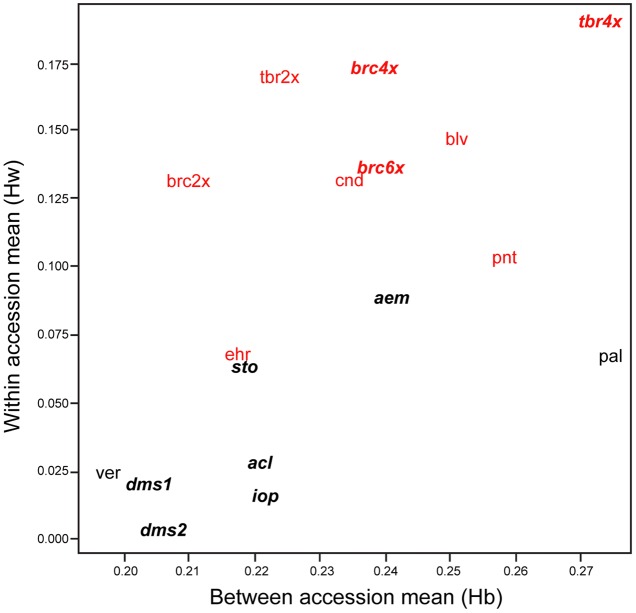
Within (*H*_w_) vs. between (*H*_b_) species diversity of the accessions examined here. Species and accession codes refer to **Table 1** and the data point lies under the center of the code. Polyploid species are shown in bold italic. Self-compatible species are shown in black and self-incompatible in red.

*^a^Species codes correspond to **Figure 1**. ^b^The two- and four-digit accessions are from the Commonwealth Potato Collection (CPC) and the six-digit accessions from the U.S. Potato Genebank (NRSP-6). ^c^N1, numbers of plants in selected accession; N2, numbers of individual plants from other accessions of same taxon. ^d^Tetraploid *S. tuberosum* Chilotanum group although strictly SC, is directly derived from SI diploids and continues to be outbred.*

### DNA Isolation

Plants were grown in the glasshouse and DNA was extracted from frozen plant leaf tissue taken from single plants using the DNeasy Plant DNA Extraction kit (Qiagen, Cat. No. 69181). Leaf material was disrupted using a MM300 Mixer Mill (Retsch GmbH and Co.) and the DNAs extracted from 2 x 96 samples simultaneously.

### AFLP Analysis

Amplified fragment length polymorphism assays were performed using a modification of the protocol, and primers used by Vos et al. (1995). The 6-bp cutting enzymes *Pst*1 and *Eco*R1, and the 4-bp cutting enzyme *Mse*1 were used. T4 DNA Ligase, T4 polynucleotide kinase, *Taq* DNA polymerase, AFLP primers and adapters were obtained individually from Invitrogen avoiding the requirement for any specific kits to be purchased. PCR reactions were set up in 384-well plates using a Beckman Biomek 2000 liquid handling device. Electrophoresis was carried out on the Biorad Sequi-Gen GT system on 5% acrylamide, 7M Urea in 1x TBE Buffer. A dual buffer system of 1x TBE and 1x TBE supplemented with 0.5M NaOAc was used to create an ionic gradient, providing improved separation of the larger fragments. A Promega *fmol* DNA Cycle Sequencing System (Promega Q4100) marker (prepared according to the protocol, but using only a d/ddT Nucleotide Mix) was run to estimate the product size and ‘control lanes’ of standard potato genotypes were included on each gel. Gels were dried onto paper exposed to X-ray film which was then developed using a Konica Minolta film processor (SRX-101A 2006).

### Gel Scoring and Data Analysis

Developed X-ray films were scanned using a standard flat-bed scanner at 300 dpi and 8 bit grayscale format. The TIFF images were scored using AFLP Quantar (Keygene Products B.V., Netherlands), which has been specifically developed for the analysis of AFLP fingerprints. Data were exported from AFLP Quantar as excel files (Supplementary Data Sheet 1).

Amplified fragment length polymorphism profiles were scored as binary matrices (1 for presence, 0 for absence of a band). Data were collated using the BioNumerics software (Applied Maths BVBA, Keistraat 120, 9830 Sint-Martens-Latem, Belgium). Analysis of genetic diversity was performed using POPGENE Version 1.32 (Yeh and Boyle, 1997). Genetic similarity measurements, clustering, and ordination were performed using Genstat version 7.2 (Lawes Agricultural Trust) and NTSYS pc 2.11L (Biostatistics Inc.).

## Results

### AFLP Data

We scored 449 polymorphic AFLP fragments from 619 plants, 313 of these representing the 17 accessions of the 13 selected taxa, the remaining 306 plants representing individual members of other available accessions of the same taxa. The scored fragments were distributed as follows among the six primer combinations: EAACMCCA (62 bands scored), EACAMCAC (79), PACMACT (96), PAGMACC (75), PATMAAC (70), PCAMAGG (67). AFLP fragment nomenclature is as follows: E(or P)_ABC_M_DEF,_ where ‘ABC’ and ‘DEF’ are the selective base extensions on the *Eco*RI(or *Pst*I) and *Mse*I primers, respectively. A UPGMA cluster analysis of a Jaccard similarity matrix based on data including representatives of many other potato taxa, was used to ensure that all individuals in this study clustered appropriately (data not shown). A separate data file containing only the AFLP markers found to be present in each taxon was generated for all taxa.

### Levels of Polymorphism within and between *Solanum* Accessions

Various measures of genetic diversity were calculated for each sampled taxon and accession. The taxon-wide statistics were calculated using a single randomly chosen individual from each available verified accession of each taxon included in the study, including the selected accession. These measures include H, Nei’s (1973) average gene diversity statistic, and I, Shannon’s Information index (Lewontin, 1972), as well as, for each chosen accession or taxon, a measure of GH as defined by Bamberg and del Rio (2004). The H, I, and GH values were found to be almost completely correlated (*r* = 0.94–1.0) with each other, and therefore, only the values of H are shown in **Table 2**, as well as information on the number and proportion of polymorphic markers in each accession and taxon.

**Table 2:** Population genetic statistics.

Taxon	Comparison	Diversity statistics^a^			Band occupancy	
			
		*N*	*H*^∗^	*N*_p_ (*P*_p_)	Acc mean no. (%)	Acc total no. (%)
**Self-incompatible** **(inc. *S. tuberosum*)**						
*S. candolleanum*	Within	207	0.14	94 (45)	152 (73)	207 (60)
	Between	336	0.24	296 (88)		
*S. ehrenbergii*	Within	130	0.07	34 (26)	111 (85)	130 (81)
	Between	140	0.22	68 (49)		
*S. boliviense*	Within	175	0.15	77 (44)	144 (82)	175 (61)
	Between	266	0.25	224 (84)		
*S. brevicaule* 4x	Within	234	0.17	125 (53)	174 (74)	234 (76)
	Between	277	0.24	185 (67)		
*S. brevicaule* 6x	Within	213	0.14	94 (44)	166 (78)	214 (69)
	Between	294	0.24	235 (80)		
*S. pinnatisectum*	Within	156	0.10	62 (40)	127 (81)	156 (76)
	Between	187	0.26	130 (70)		
*S. brevicaule* 2x	Within	179	0.14	89 (50)	133 (81)	165 (50)
	Between	319	0.21	279 (87)		
*S. tuberosum* L. Andigenum group (2x)	Within	225	0.17	144 (64)	153 (70)	219 (53)
	Between	401	0.23	368 (92)		
*S. tuberosum* Chilotanum group (4x)	Within	289	0.19	183 (63)	199 (69)	290 (81)
	Between	332	0.27	270 (81)		
**Self-compatible**						
*S. acaule*	Within	183	0.03	17 (9)	173 (95)	183 (60)
	Between	288	0.22	226 (78)		
*S. ×aemulans*	Within	196	0.09	58 (30)	171 (87)	196 (69)
	Between	265	0.24	190 (72)		
*S. demissum* (27)	Within	213	0.02	19 (9)	194 (91)	213 (58)
	Between	359	0.20	310 (86)		
*S. demissum* (3850)	Within	196	0.01	4 (2)	191 (97)	196 (47)
	Between	368	0.20	319 (87)		
*S. iopetalum*	Within	201	0.01	13 (7)	192 (96)	201 (63)
	Between	311	0.22	225 (72)		
*S. palustre*	Within	134	0.07	29 (22)	117 (87)	135 (55)
	Between	229	0.27	179 (78)		
*S. stoloniferum*	Within	199	0.06	75 (38)	181 (89)	204 (51)
	Between	402	0.22	370 (92)		
*S. verrucosum*	Within	146	0.02	20 (14)	132 (90)	146 (51)
	Between	282	0.20	222 (79)		


*^a^*N*, number of AFLP fragments observed; ^∗^*H*, Nei’s (1973) gene diversity; *N*_*p*_, number of polymorphic AFLP fragments; *P*_*p*_, percentage of polymorphic AFLP fragments.*

Our data show that the sampled accessions of SC potato species show considerably lower levels of AFLP diversity than accessions of SI taxa. The level of within-accession average expected heterozygosity (*H*_w_) (Nei, 1973) ranges from 0.01 to 0.09 (mean = 0.04) for SC taxa and from 0.07 to 0.19 (mean = 0.14) for SI taxa (**Figure 1**), a more than three-fold difference (*t* = 6.26, *P* < 0.001). The three accessions of the allohexaploid 4EBN *S. demissum* and *S. iopetalum* are extremely lacking in diversity, as are, to a lesser extent, the diploid 2EBN *S. verrucosum* and the allotetraploid 2EBN *S. acaule*. Another allotetraploid, *S. stoloniferum* shows considerably more diversity than *S. acaule*. The allopolyploid 2EBN *S.* ×*aemulans* is an outlier among the SC species, showing an unusually high level of diversity (0.09), more akin to that of some of the SI taxa sampled. The most diverse diploid accession of an inbreeding taxon is *S. palustre* (diploid 1EBN), which is more diverse than the Mexican diploid 1EBN SI species *S. ehrenbergii*. A second SI Mexican species *S. pinnatisectum* (also diploid 1EBN) shows levels of diversity similar to the more diverse SC species. The diploid 2EBN wild outbreeders (*S. boliviense*, *S. brevicaule*, and *S. candolleanum*) show very consistent levels of within-accession average diversity (0.14–0.15) whereas the SI diploid 2EBN cultivated *S. tuberosum* Andigenum group (2x) is more diverse (0.17). The polyploid accessions of *S. brevicaule* (both 4x and 6x populations) shows relatively high levels of genetic diversity (0.17 and 0.14 for tetraploid and hexaploid, respectively). The autotetraploid cultivated 4EBN *S. tuberosum* Chilotanum group (4x) is the most diverse (0.19) of the accessions sampled in this study.

The levels of between accession diversity (*H*_b_), determined by using a single individual from each available accession, are more consistent, varying from 0.20 to 0.27 (mean = 0.22) for inbreeding accessions and from 0.21 to 0.27 (mean = 0.24) for outbreeding accessions from SI species. The difference in mean diversity values for SI and SC accessions is not statistically significant (*t* = 1.77, *P* > 0.05). The inbreeders are generally only very slightly less diverse than the outbreeders at the taxon-wide level, although *S. verrucosum* and *S. demissum* appear to possess less genetic variability. The self-compatible species *S. palustre* appears to be a very diverse taxon but is represented by only six samples. Despite its wide range of morphological variation (Hawkes, 1990), the self-incompatible species *S. brevicaule* 2x shows a relatively low level of ‘between-accession’ variation across the 24 populations sampled. The overall correlation between the within and between accession diversity values is 0.61, but this is largely due to a significant correlation between the two mean diversity measures for SC accessions (*r* = 0.76); the figure for the SI accessions being non-significant (*r* = 0.34, *P* > 0.05).

### Relationships between Individuals in a Single Accession to Individuals from Different Accessions of the Same Species

**FIGURE 2:**
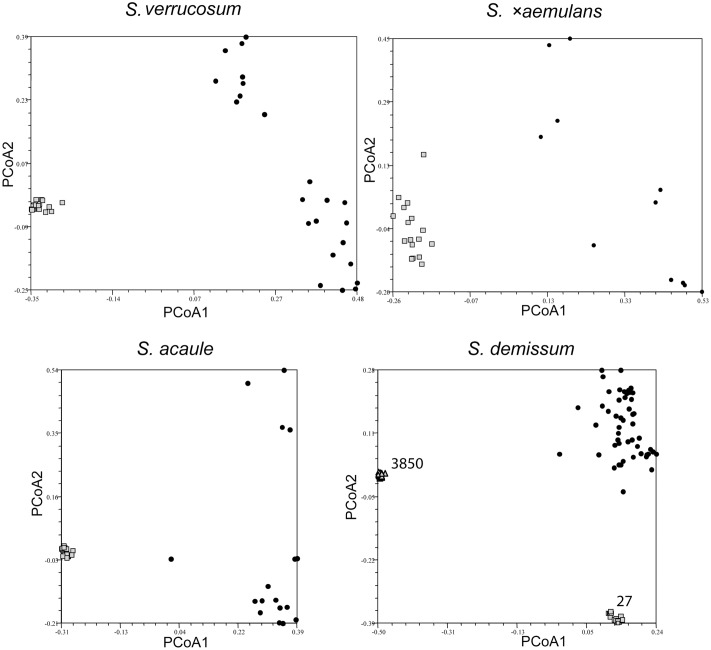
Principal coordinate analysis showing clustering of individuals in single genebank accessions of self-compatible species *Solanum verrucosum*, *S. acaule*, *S.* ×*aemulans*, *S. demissum* relative to a broader species sample, represented by single individuals from several genebank accessions. The relevant accessions are shown as lightly shaded (squares and triangles); the individuals representing the taxon as black circles.

**FIGURE 3:**
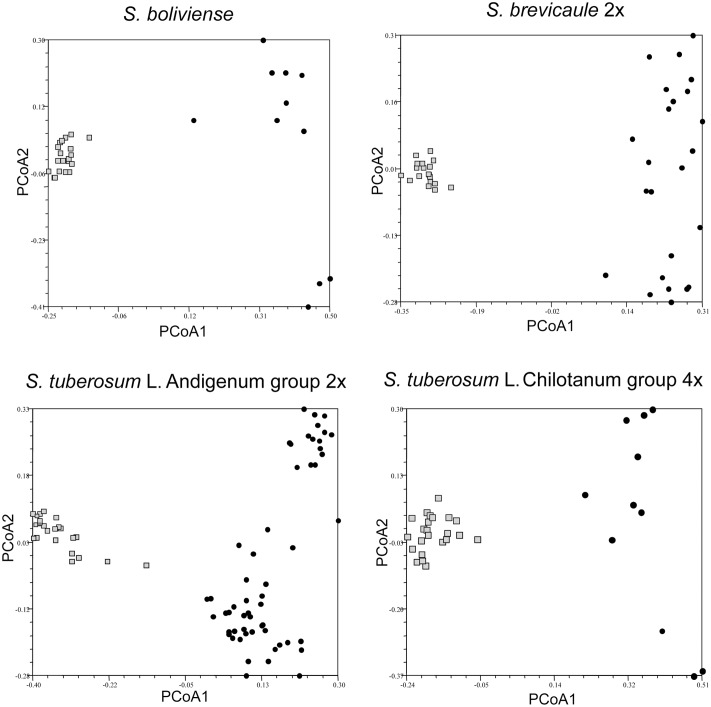
Principal coordinate analysis showing clustering of individuals in single genebank accessions of self-incompatible species *Solanum boliviense*, *S. brevicaule* 2x, *S. tuberosum* Andigenum group (2x), *S. tuberosum* Chilotanum group (4x) relative to a broader species sample, represented by single individuals from several genebank accessions. The relevant accessions are shown as lightly shaded (squares and triangles); the individuals representing the taxon as black circles.

**FIGURE 4:**
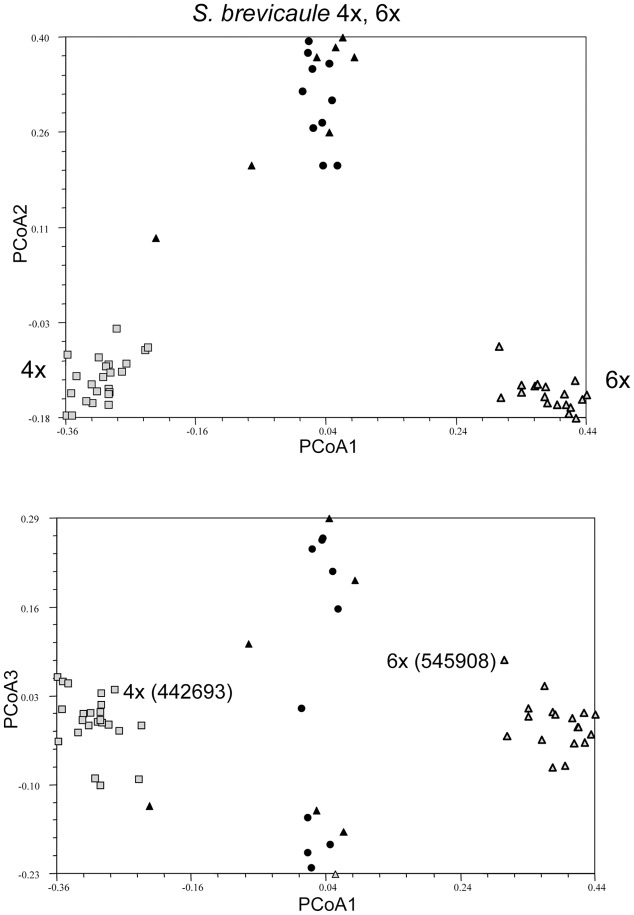
Principal coordinate analysis showing clustering of individuals in single genebank accessions of probable self-incompatible species *Solanum brevicaule* 4x and 6x showing clustering of two accessions differing in ploidy relative to a broader species sample, represented by single individuals from several genebank accessions. The tetraploid accession members are denoted by gray shaded squares, hexaploid accession members are by gray shaded triangles, and the taxon members by black circles (4x) or triangles (6x) depending on ploidy.

The AFLP marker data for each taxon were used to generate ‘Jaccard’ similarity matrices, which were subjected to a principal coordinate analysis (PCoA) to allow graphical visualization of the relationships among plants in each sampled accession relative to those representing the same taxon. **Figures 2**–**4** show the PCoA plots for nine representative taxa (**Figure 2**: SC taxa *S. verrucosum*, *S.* ×*aemulans*, *S. acaule*, *S. demissum*; **Figure 3**: SI taxa *S. boliviense*, *S. brevicaule* 2x, *S. tuberosum* Andigenum and Chilotanum groups; **Figure 4**: the polyploid putative outbreeder *S. brevicaule* 4x, 6x, with two panels showing PCoA coordinates 1 vs. 2 and 1 vs. 3). **Figure 2** shows clearly both the high degree of clustering among individuals from each accession of self-compatible species, as well as the relatively large genetic separation between any such individual and individuals from other accessions of the same taxon. This tight clustering (except *S.* ×*aemulans*) suggests that any single plant is highly representative of the accession from which it is drawn (**Figure 2**). In the case of *S. demissum*, where two accessions are sampled, each accession is clearly separated from each other (both axes), and from the general ‘population’ (by either the first or second PCoA axis). It is interesting to note, however, that the self-compatible taxon *S.* ×*aemulans* shows a much more dispersed pattern than the other inbreeders shown, in line with the somewhat higher level of intra-accession variability shown in this species.

For the SI taxa it is clear that there is considerably less within-accession clustering (**Figure 3**). In all cases the first PCoA clearly separates the individuals in each accession from the individuals representing other accessions of the same taxon. However, the accession of *S. tuberosum* Andigenum group (2x) contains two individuals that would be considered outliers, suggesting that highly outbred populations may require deeper sampling. If all of the individuals representing the species *S. brevicaule*, regardless of ploidy, are considered as a single group the PCoA analysis of the resulting Jaccard similarity matrix reveals interesting relationships among the tetraploid and hexaploid individuals present (**Figure 4**). The first PCoA clearly separates the two sampled accessions (one 4x, one 6x), and the second PCoA separates these from the other individuals representing this taxon. The third PCoA separates the other individuals representing the species (i.e., not present in the two sampled accessions) into two groups which do not cluster according to ploidy level, suggesting that there is a genetic structure to the *S. brevicaule* 4x and 6x material that is not related to ploidy differences *per se*. This suggests recent gene flow between populations differing in ploidy.

### Frequency of AFLP Marker Alleles within Each Population and Taxon

For each population and taxon the mean frequencies and total numbers of each individual AFLP marker were calculated (**Table 2**). These figures suggest that any randomly selected plant contains ∼91% (range 87–97%) and 77% (range 69–85%), respectively, of marker bands present in the parent accession for SC and SI accessions, respectively (*t* = 5.93, *P* < 0.001). The same type of calculation was used to express the proportion of marker fragments present in each taxon that were contained in each of the sampled accessions. These figures average 57% (range 47–69%) and 67% (range 49–81%) for SC and SI taxa, respectively (**Table 2**), the difference in means being very close to statistical significance (*t* = 2.12, *P*∼0.05). These results show that an individual plant is considerably more representative of an inbred than an outbred accession in terms of the proportion of marker alleles that it contains. In contrast, an accession of an outbreeding species appears to better represent the marker allele complement in the species as a whole than does an inbreeding accession, although there is a wide range of variation in these data.

**FIGURE 5:**
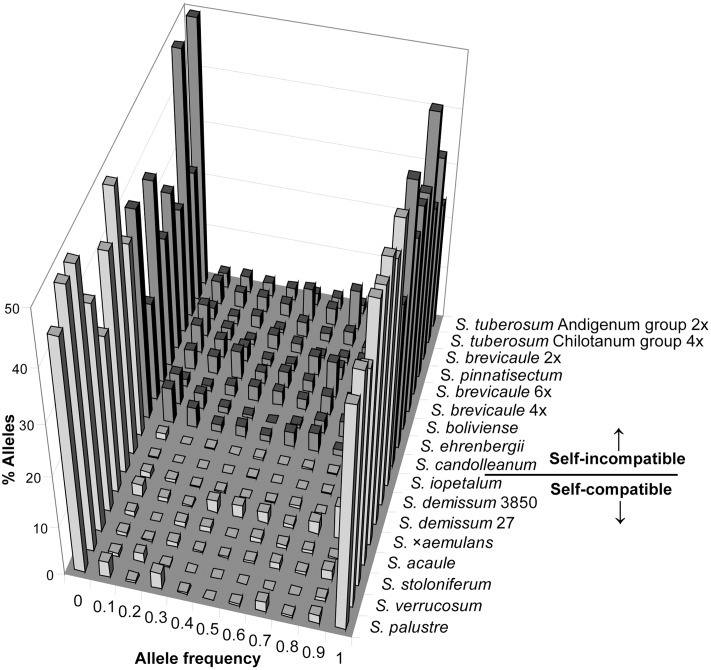
AFLP allele frequency distributions grouped by self-compatible and self-incompatible species. **Table 1** lists accessions and variant accession numbers.

By way of illustration, a plant drawn from *S. demissum* accession 3850 contains 97% of the band total for that accession. Similarly the mean allele composition of the *S. acaule* and *S. iopetalum* accessions are over 95%. For the highly outbred cultivated accessions *S. tuberosum* Andigenum group and *S. tuberosum* Chilotanum group a plant contains 70% and 69% of alleles, respectively. In terms of outbred accessions representation of species allelic composition, *S. ehrenbergii* and *S. tuberosum* contain the greatest degree of species composition (81% for both). *Solanum ehrenbergii* possess relatively low intra- and inter-accession diversity whereas *S. tuberosum* has high levels of both intra- and inter-accession diversity. For the inbreeding taxa the allopolyploid accessions contain a higher proportion of the total species complement than the diploids, although one of the *S. demissum* accessions (3850) contains a very low proportion (47%), possibly due to it being extremely low in genetic diversity and genetically dissimilar to other accessions of this species. The same is true for the polyploid outbreeding taxa but to a slightly lesser degree. The AFLP allele frequency distributions for each accession are shown in **Figure 5**. It is clear that the self-compatible accessions contain very few alleles at intermediate frequencies (0.1–0.9), whereas self-incompatible accessions contain a somewhat greater proportion of alleles at such frequencies, although the proportion of ‘fixed’ alleles is high in both cases.

## Discussion

### How Effective Is AFLP as a Measure of Population Diversity?

We have used the highly multiplex and repeatable AFLP method for assessing genetic diversity in genebank accessions of potato. AFLP is a dominant marker system, and as such precludes the direct identification of heterozygotes and calculation of heterozygote frequencies. AFLP replaced RAPD technology as well as other dominant marker technology as the method for fingerprinting genebank material without knowledge of prior sequence information and is a technique which has been used for diversity studies in a wide range of organisms (Bensch and Akesson, 2005). Other methodologies, such as simple sequence repeat (SSR) and single nucleotide polymorphism (SNP) markers, have some advantages for this purpose. For potato there exist a large number of SSR markers (Ghislain et al., 2004) that are extremely useful for linkage and diversity analysis but these are not amenable to high throughput deployment across a large set of taxonomically diverse plant material. Moreover, SSR markers require absolute conservation of flanking primer sequences and this is far less likely across taxonomically distant gene bank material such as those studied here. SNP markers have been used in the analysis of plant germplasm, for example the use of such markers in cassava reported by De Oliveira et al. (2014). However, the high cost, relative lack of flexibility, and likely ascertainment bias when using a SNP platform, such as the potato 8303 SNP marker platform reported in Felcher et al. (2012) does not make them a viable choice for this type of analysis. The use of individually assayed SNPs (e.g., using SNP-CAPS or some proprietary method such as KASP) suffers from the same shortcomings as use of SSR markers (i.e., low number of loci sampled). Recently, Elshire et al. (2011) reported the use of ‘genotyping by sequencing’ (GBS) for genotyping in high diversity species. While this approach is a cost-effective option for crop germplasm studies, the reliance on high throughput sequencing data and significant downstream bioinformatic analysis renders it unsuitable for use in most genebank studies. AFLP markers present their own challenges to deployment; this experiment required scoring of AFLP profiles across several sequencing gels. Band identity was aided by the use of both sequencing ladder markers and standard potato genotypes, reducing the risk of mis-classifying apparently co-migrating markers that may differ by a single base pair. True co-migrating AFLP alleles which are not allelic could confound the analysis, but, in similar and more diverse germplasm AFLP has been found to be suitable for examining taxonomic relationships (Kardolus et al., 1998; McGregor et al., 2002; Spooner et al., 2005). It is clear that, despite the deficiencies of AFLPs due to their dominant nature, these are somewhat offset by the high numbers of markers that can be examined with relatively small effort, particularly across a taxonomically diverse group where polymorphic locus-specific markers such as SSRs may fail or give rise to undetected null alleles. In this study, we examined 449 AFLP loci through deployment of six primer combinations, an average of ∼75 per primer combination. It is also important to consider the flexibility of AFLP markers for this type of germplasm analysis. More recently developed SNP platforms require the consolidation of material to provide a certain number of assays (48, 96, 384, etc.) for cost effective deployment. AFLPs are more flexible for use by germplasm curators and are perfectly suited for the analysis of diversity within and between genebank accessions.

### Levels of Intra-accession vs. Inter-accession Diversity

One of the primary aims of this study was to establish the relative levels of within and between accession AFLP diversity for a series of potato taxa representing most of the known common breeding systems and ploidy levels in the tuber-bearing (and a close relative non-tuber-bearing) members of the genus *Solanum*. We decided on the use of a single well-described genebank accession to represent a population for each taxon, and a single individual from each of several genebank accessions to represent each taxon. Our data show that genebank accessions of inbreeding, SC *Solanum* species show considerably lower levels of gene diversity than accessions of outbreeding SI taxa. The measure we have used (Nei, 1973) correlates extremely closely with other types of diversity statistics calculated from these data. Allopolyploid populations are particularly lacking in gene diversity, with the exception of *S. ×aemulans*, which contains an unusually high level of average heterozygosity, approaching that of some of the outbreeding populations sampled. Of the outbreeding SI types the diploid species *S. ehrenbergii* is less diverse than some inbred populations, and *S. pinnatisectum* shows similar levels of diversity to the more diverse inbreeding taxa. The diploid wild outbreeding taxa examined here (*S. boliviense*, *S. brevicaule*, *S. candolleanum*) show very consistent levels of within-population average diversity. The diploid and tetraploid cultivated accessions (*S. tuberosum* Andigenum group 2x and *S. tuberosum* Chilotanum group) are the most diverse in this study. It is interesting that, despite the derivation of accessions from single parental clones, the autotetraploid cultivated *S. tuberosum* accession sampled is the most diverse. Highly heterozygous cultivated tetraploid material appears to maintain even higher levels of diversity in single clones than is found in SI wild species. In general, the SI taxa show highly consistent levels of within-accession polymorphism, except the diploids *S. pinnatissectum* and S. *ehrenbergii*, which show lower levels of within-accession polymorphism. Of particular note is the high level of within accession polymorphism shown by the polyploid, presumed SI, species *S. brevicaule* (both 4x and 6x populations), whose mating behavior is less well understood than those of the other polyploids examined. The former identifications for the accessions here examined for *S. brevicaule* 4x and 6x was *S. oplocense*, which was considered a SC species (Ashley and Hawkes, 1979; Chavez et al., 1988; Hawkes and Hjerting, 1989), but this was based on few experiments and inconsistent results. We here group these polyploid accessions of *S. brevicaule* as a tentative self-incompatible species based on the AFLP frequency distributions as shown in **Figure 5**, but the compatibility of these cytotypes is in need of further study.

The pattern of AFLP variation in the SC taxa in this study suggests that the levels of inbreeding in these taxa are variable. *Solanum palustre* retains intra-accession diversity at higher levels than that of many of the SC polyploid taxa, and this implies that inbreeding is only partial in this species. There may be specific mechanisms to promote outcrossing in this SC species. In addition to these features of taxa already known to be SC, the low diversity found in the two Mexican diploid species mentioned above could be due to a loss or suppression of the SI system in these accessions, or simply a bottleneck prior to or during capture as a genebank accession.

The levels of between-population variability, as determined using a single individual from each available population, are more consistent with SC species being only very slightly less diverse than the SI species at the taxon-wide level. The SC *S. palustre* appears to be a very diverse taxon but is represented by only six individuals. The SI species *S. brevicaule* 2x shows relatively low levels of between-accession variation at the DNA level, and this suggests that the species may have recently radiated to generate the extensive morphological variation known for this taxon. Of course the results obtained for each taxon are influenced to some extent by the diversity of the accessions present in the genebank, not necessarily a measure of true taxon diversity, for example if accessions were only sampled from a narrow geographic range of a species overall range.

Amplified fragment length polymorphism has been deployed recently to assess levels of diversity and systematic relationships in and among 12 wild species of *Solanum* section *Basarthrum* (Prohens et al., 2006), where inferences were made on autogamy and allogamy from patterns of AFLP diversity. These authors calculated species gene diversity values ranging from 0.023 to 0.159, and they conclude that there are not large differences in gene diversities between SC and SI species. Our data would suggest otherwise, although it is possible that genebank maintenance, with no or very little possibility for population admixture or migration, has ‘reinforced’ natural differences due to SI and SC mating systems. Our results can also be compared to earlier studies using allozymes and other markers. The comprehensive survey by Hamrick and Godt (1989) has examined plant allozyme diversity at population and species level in comparison to life history and other traits (geographic range, successional status, etc.) in 449 species from 165 genera. They reported that, at the population level, the plant breeding system and geographic range accounted for the largest proportion of variation in genetic diversity. They also suggest that population-level diversity accurately reflects species-level diversity. In this study we find that this is truer of SC than SI taxa. Hamrick and Godt (1989) provide average diversity statistics at both population and species level. Selfers show diversity values of 0.124 (species) and 0.074 (population), as compared to 0.22 and 0.04 in this study. The values for outcrosses in the previous study are presented separately for wind (0.162 and 0.148) and animal (0.167 and 0.124) pollinated taxa. These compare to average diversity values of 0.24 and 0.14 for SI taxa in this study. As we have sampled a single plant from each accession to estimate taxon-wide diversity, it is likely that our figures are over-estimates of the true values. Schoen and Brown (1991) published another summary of allozyme data for a wide array of plant genera, including *Arabidopsis, Hordeum, Phaseolus*, and *Pinus* species. In the published survey, the average within population gene diversities are 0.125 (inbreeding taxa) and 0.257 (outcrossing taxa), as compared to 0.04 and 0.14 in this study. These data suggest, perhaps as would be expected, that AFLPs underestimate gene diversity with respect to allozymes, although the mean diversities in Schoen and Brown (1991) are considerably higher than in the earlier study. The fact that this study shows a higher degree of difference between outcrossing and inbreeding taxa is perhaps due to the fact that we have sampled from within a single genus (i.e., tuber-bearing *Solanum* species) rather than an assortment of genera in the cited reviews. Schoen and Brown (1991) also document the tendency of inbreeding taxa to show more variation in average gene diversities across different populations. They interpret these findings as suggestive of a need to survey populations of inbreeding taxa for molecular diversity to allow informed decisions about how such taxa should be represented in genebanks. For outbreeding taxa they suggest that, as there is less variability between populations, there is less of a need for such surveys.

### Average Similarities of Individuals in Genebank Populations

This analysis seeks to address the question of how representative is any randomly selected plant of an accession for a particular type of breeding system, ploidy or EBN. We have calculated similarity matrices for each of the taxa in this study. The relationships between the individual plants representing each accession and taxon have been visualized using ordination approaches, specifically PCoA plots derived from the similarity matrices for each selected taxon. In most cases, the taxa behave as we would expect from their breeding systems and fit in with the diversity measurements reported. A second approach has been to examine the numbers and proportions of AFLP markers present in individual plants and accessions relative to the parental accession and taxon. In making observations regarding the extent to which plants or accessions are representative of the population or taxon from which they come we are equating the shared presence of an AFLP allele as being a meaningful, informative character state (and analogous to shared presence of an allele of a gene) as compared to shared AFLP band absences, which are assumed to represent many different possible character states. These data, taken in combination with the diversity statistics, suggest that an individual plant of an inbred or slightly outbred accession is highly representative of its parental accession and that use of a single plant to represent such populations is permissible for all but the most detailed of taxonomic studies. These conclusions appear to be particularly applicable to allopolyploid inbred species, accessions of which appear to extremely low in genetic diversity. However, as some SC species have higher levels of intra-accession diversity, one strategy for maintaining all SC *Solanum* genebank accessions would not be appropriate.

For outbreeding SI taxa the data are more equivocal. It is not possible, from the results of this preliminary study, to state exactly how many plants are required to sample fully the genetic diversity of an outbred SI population. However, it is clear that individual highly outbred *Solanum* populations, such as the cultivated accessions examined here, are extremely useful reservoirs of genetic diversity in genebanks. One conclusion from this study is that genebanks should try to maintain large numbers of accessions for inbreeding taxa, particularly the allopolyploids, but that relatively few well-selected highly diverse accessions can be used to represent outbreeding taxa. A second conclusion is that the numbers of plants raised for seed increase of inbreeding taxa are less critical than for outbreeders, individual populations of which are more susceptible to the erosion of genetic diversity through enforced inbreeding, although the lower limits to the number of plants used will differ according to the level of diversity in the accession.

These results have practical applications for the use of genebanks for identification of novel trait variation, such as novel sources of pest and disease resistance. Our results suggest that many inbreeding accessions will be more or less fixed for such resistances if present in the accession, whereas for more outbred accessions significant sampling may be required prior to use of such material in the development of mapping work and introgression into adapted populations.

## Author Contributions

DS, GB, and RW conceived of the topic, designed the experiments, and wrote the paper. KM performed the experiments and analyzed the data.

## Conflict of Interest Statement

The authors declare that the research was conducted in the absence of any commercial or financial relationships that could be construed as a potential conflict of interest.
